# Retinal Prostheses and Artificial Vision

**DOI:** 10.4274/tjo.galenos.2019.44270

**Published:** 2019-09-03

**Authors:** Emin Özmert, Umut Arslan

**Affiliations:** 1 Ankara University Faculty of Medicine, Department of Ophthalmology, Divisions of Medical and Surgical-Retina-Bionic Eye and Artificial Vision, Ankara, Turkey; 2 Bio-Retina Eye Clinic, Ankara, Turkey

**Keywords:** Artificial vision, bionic eye, visual prosthesis, Argus II, retinal prosthesis, outer retinal degeneration, retinitis pigmentosa, phosphene

## Abstract

In outer retinal degenerative diseases such as retinitis pigmentosa, choroideremia, and geographic atrophy, 30% of the ganglion cell layer in the macula remains intact. With subretinal and epiretinal prostheses, these inner retinal cells are stimulated with controlled electrical current by either a microphotodiode placed in the subretinal area or a microelectrode array tacked to the epiretinal region. As the patient learns to interpret the resulting phosphene patterns created in the brain through special rehabilitation exercises, their orientation, mobility, and quality of life increase. Implants that stimulate the lateral geniculate nucleus or visual cortex are currently being studied for diseases in which the ganglion cells and optic nerve are completely destroyed.

## Introduction

Nearly half of visual impairment worldwide is caused by retinal diseases. Degenerative retinal diseases such as retinitis pigmentosa (RP), choroideremia, and age-related macular degeneration (AMD) begin in the outer retinal layers and progress gradually, with the inner retinal layers remaining largely unaffected until advanced stages of disease. Histopathological studies have shown that 70% of photoreceptors are lost in AMD, while 93% of the retinal ganglion cells (RGCs) survive. RP mainly affects the photoreceptor layer; in the macular region, 78-88% of the inner nuclear layer (INL) and approximately 30% of the ganglion cell layer (GCL) remains intact. RP leads to cell loss in all retinal layers in the extramacular region; the INL and GCL are relatively less preserved than the macular region. There is no difference between the different genetic types of RP in terms of macular cell loss. In the extramacular region, more cells are preserved in autosomal-dominant RP.^1,2,3^

Neovascular AMD is treated at significant rates using intravitreal anti-VEGF drug injections, but there is not yet a proven effective treatment for geographic atrophy (GA), an advanced stage of dry AMD. RPE65 gene therapy and slow-release ciliary neurotrophic factor implants are being used in the treatment of RP, and stem cell research is ongoing. However, there is no proven, definite treatment approach. Retinal prostheses developed in recent years are promising for eyes with severe visual impairment due to outer retinal degeneration.^2,4^

## Electrical Stimulation of the Retina

The neural network in the inner retina, which is relatively unaffected by the degenerative process, is electrically stimulated in a controlled manner with microelectrode arrays placed under the retina or on the macula. Spatial visual perception can be generated with simultaneous pattern stimulation of multiple retinal locations. Action potentials generated in the retinal ganglion cells are relayed to the brain through the intact optic nerve and optic tracts and perceived as phosphenes; these can be localized based on the stimulated retinal field. Phosphenes are generally seen as white, round or oval spots of light that are of varying size and cause no discomfort.^2,5^

Current research and development on visual prostheses and artificial vision is mainly focused on retinal implants (epiretinal, subretinal, suprachoroidal) and cortical implants. Retinal implants are being investigated for diseases such as RP, choroideremia, and GA, which cause degeneration of the outer retinal layers. Cortical implants placed in the primary visual cortex are being investigated as an option for patients who have completely lost their vision for various reasons.^6^

Microelectrode arrays placed in the subretinal space act as a phototransducer and are in a more natural position, like an artificial photoreceptor layer; however, the surgical and technical problems associated with them have not been totally solved.^7^ Prostheses placed in the suprachoroidal space through a scleral incision stimulate the bipolar cells in the outer retinal layer indirectly via the choroidal layer, without touching the degenerated retinal tissue.^2^

## A. Subretinal Implant

This retinal prosthesis is comprised of light-sensitive microphotodiode arrays. The array is placed under the retina, and is therefore in a more physiological position between the retinal pigment epithelium and degenerated photoreceptor layer. Placement of the implant in the subretinal space is done either externally via a scleral incision or internally via vitrectomy and retinotomy. The microphotodiode array consists of thousands of small, light-sensitive units, each comprising a diode, amplifier, and microelectrode components, which are embedded in a silicone matrix. Microelectrodes made of titanium nitrite and gold are in contact with the relatively intact retinal neural network. This array receives light, processes and amplifies the signal, and stimulates the nerve cells. The photovoltage process that occurs when crystal is exposed to natural light entering the eye generates an electric current that directly and precisely stimulates the degenerate photoreceptors and bipolar cells it contacts. These processes are carried out by the intact residual inner retinal layers; therefore, a small threshold is enough to generate a visual response. However, natural light cannot provide enough energy to the light-sensitive microphotodiode array, so power must be supplied by external electronics. Therefore, an induction coil is embedded subcutaneously in the retro-auricular region and power is supplied to the microphotodiode array through a cable. This implant has certain advantages: It provides a more physiological form of stimulation and implantation of the array into the submacular region is easy. Subretinal implants use the patient’s own optic system; therefore, an external camera or external image processing unit is not required to capture images. Existing eye movements and gaze are sufficient for object localization, and scanning head movements are not necessary like with epiretinal implants. The Alpha-IMS is a 1500-pixel subretinal implant that obtained CE mark approval in Europe in 2013.^2,7^

## B. Epiretinal Implant

Epiretinal implants do not have microphotodiode units that generate electric current via natural light, like those in subretinal implants. Visual information collected by a microcamera mounted on removable glasses is processed by a video processor and encoded as spatial electrical potential patterns that directly stimulate the ganglion cells and nerve fibers. Because the stimulated area is large, it generates indistinct malformed phosphenes whose shapes are corrected through electronic processing. As this system bypasses the residual inner retinal layers and directly stimulates ganglion cells, it requires complex image processing techniques with electronic circuitry instead of neural network processing.^2,8^

Epiretinal prostheses are not useful for vision losses associated with glaucoma and optic nerve pathologies in which retinal ganglion cells and axons are damaged. The optic nerve, lateral geniculate nucleus, and visual cortex can also be used as targets of stimulation in complete vision loss due to any cause. Human trials of visual cortical stimulators were initiated in 2017 (ORION project, Second Sight, Sylmar).^2,6,9^

There are ongoing studies of different epiretinal implant designs in various centers. However, the first and currently only product to receive both Food and Drug Administration (2013) and CE mark (2011) commercial approval is the Argus^®^ II epiretinal prosthesis system (Second Sight Medical Products Inc., Sylmar, CA, USA).^3^

## Argus II Epiretinal Prosthesis

Initial research into this prosthesis began in 1990. Following feasibility studies of the 16-electrode Argus I initiated in 2002, prospective multicenter studies with the 60-electrode Argus II were initiated in 2007. Biocompatibility, reliability, and benefit to the patient has been demonstrated, and it is now the most implanted visual prosthesis in many countries, including Turkey.^3^

The Argus II system uses an epiretinal approach, with the microelectrode array implanted into the inner macular surface, very close to the nerve fiber layer. Thus, direct and controlled electrical stimulation is applied to the partially functional inner retinal neural network, RGCs, and/or bipolar cells. The action potential formed by these inner retinal cells travels to the visual cortex via the optic nerve and optic tracts to generate a basic visual perception called a phosphene.^10^

## 1. System Components

**A. Removable external component:** Consists of two parts:

- Glasses the patient can wear and remove: A video microcamera is mounted in the bridge and an external connection coil is attached to the sidearm. The transmitter coil sends external power and incoming information from the video processing unit (VPU) to the implanted coil wirelessly via radiofrequency.

- The VPU is a portable computer the patient can mount on their belt or carry in their pocket.

**Figure 1:**
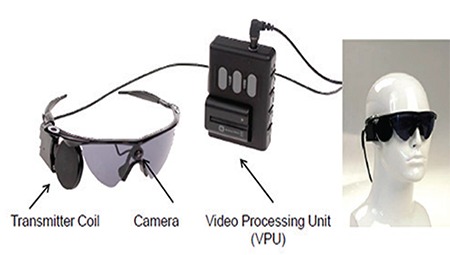
The removable external part of the Argus II epiretinal prosthesis system^13^

When the patient puts on the glasses and switches on the system, the video microcamera receives images as the patient makes scanning head movements, and it sends these to the VPU via cable. The image processor reduces the images’ resolution and converts them into electrical signals in real time to produce digital stimuli. A series of stimulation commands are generated. The VPU is custom programmed and cannot be used by other patients. Ultimately, visual images are converted into a template of electrical stimulations, which are sent to the external transmitter coil via the same cable. These signals are then transmitted wirelessly to the receiver coil sutured to the sclera via radiofrequency connection (Figure 1).

**B. The permanent ocular implant component:** The implanted part of the Argus II comprises the following interrelated electronic components that are mounted on a material similar to the silicone scleral band used in retinal detachment operations:

**- Receiver coil: **Following dissection of the conjunctiva and Tenon’s capsule, the receiver coil is positioned under the lateral rectus muscle and then sutured to the sclera. It wirelessly receives electric power and visual signal information from the external transmitter coil at the sidearm of the glasses. The electrical power supplies the microelectrodes and electronic circuits that deliver controlled stimulation to the retina to produce a visual image.

**- Electronics case: **This component is sutured to the sclera and connected via electronic cable to the microelectrode array that will be tacked to the macular surface. Using its decoding circuitry, it decodes commands encoded in the radiofrequency signals. According to these commands, the necessary pixelated stimulation output is generated and sent to the intraocular microelectrode array.

**Figure 2:**
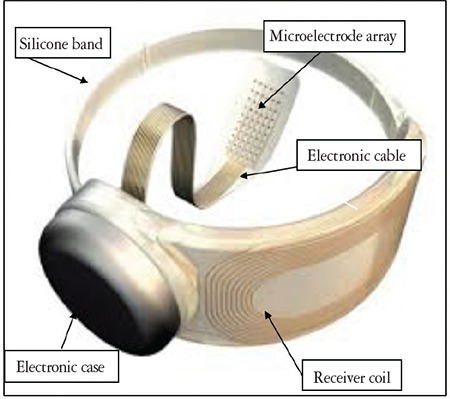
The permanent ocular implant part of the Argus II epiretinal prosthesis: electronics case and receiving coil on a silicone band, electronic cable, and microelectrode array^13^

**Figure 3:**
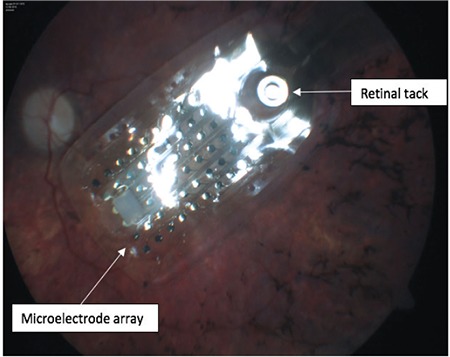
Argus II epiretinal prosthesis with a 60-electrode array positioned on the macula and attached to the sclera with the retinal tack piercing the choroid (surgery performed by E.Ö.)

**Figure 4:**
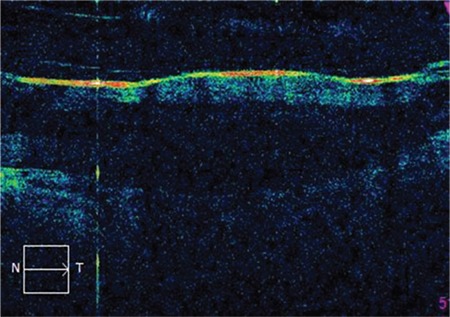
Cross-sectional spectral-domain optical coherence tomography image of the microelectrode array placed on the inner surface of the macula (surgery performed by E.Ö.)

**- Microelectrode array:** Located at the end of the electronic cable, it is inserted into the vitrectomized vitreous cavity through a 5.2-mm pars plana incision. This array comprises 60 independently active platinum microelectrodes organized in 6x10 grid embedded in waterproof silicone matrix. The dimensions of the array are 4.7x7.1 mm (Figure 2). The relatively intact neural network in the inner macular surface is stimulated by the spatial and temporal stimulation patterns transmitted to the array (Figures 3 and 4). The resulting action potentials are relayed to the brain via the optic nerve tract formed by RGC axons, generating visual perception in the form of an array of light spots (phosphenes).^3,7,11^

The electronic components constituting the Argus II system are compatible with magnetic resonance imaging (MRI) up to a field strength of 3 Tesla (glasses and VPU must be removed during imaging). However, the implanted components generate a 50x50 mm image artifact in MRI images, making it difficult to examine orbital structures.^3^

## 2. Stimulation Strategies

The diameter of each electrode in the array is 200 µm, while the RGC bodies are 15 µm in diameter. Therefore, every electrode stimulates many RGCs together with their axons in the neural network it contacts. Thus, the generated phosphenes are not punctate but linear in shape, a factor that compromises the clarity of the image. Various stimulation strategies have been developed to prevent the formation of linear phosphenes:

- Varied current-controlled stimulation pulses are used for electrical stimulation. Of these, the use of a charge-balanced, biphasic, cathodal first pulse with zero net charge injection enables the stimulation of more ganglion cell bodies without retinal damage. When the cells bodies are stimulated, the threshold for phosphene generation is lowered, and a greater number of small, punctate phosphenes are generated.

- With the bipolar stimulation technique, in which one of the epimacular array electrodes is used as a return electrode, the stimulated retinal field is smaller and therefore the probability of generating small punctate phosphenes increases.

- Acceptable patient mobility can be achieved with about 600 electrodes; however, this requires a reduction in electrode diameter. As the electrode’s surface area is reduced, current density and charge density increase rapidly, and the electrochemical reactions that occur lead to retinal tissue damage. In order to increase the number of pixels without changing the existing hardware in Argus II, stimulation strategy software such as “current focusing” and “current steering/virtual electrode” is being developed. With these virtual applications it is now possible to make 209 tiny electrical stimulations, which increases the likelihood of being able to stimulate fewer RGCs and to stimulate their bodies in particular. The ability to generate a greater number of smaller punctate phosphenes will enhance the clarity of the image.^2,12^

## 3. Stages of Argus II Implantation- Selecting suitable candidates

Patients should be in fair medical and psychological condition and be motivated. The patient’s expectations from the surgery should be determined, and they should fully understand the benefits and limitations of implantation. Both the patient and their family members should be ready and capable of carrying out the long and challenging process ahead;

- Special preoperative training of the surgeon, medical personnel, and technical staff about the implantation;

- Introducing the patients to the system components and instructing them on their use;

- Procuring and preparing specialized surgical materials and making checklists;

- Performing the implantation procedure with special technical support;

- Closely following the patient postoperatively for conjunctival erosion/dehiscence, endophthalmia, severe hypotonia, choroidal/retinal detachment, and array position;

- Performing the calibration (fitting) procedure 15 days after implantation: The electrodes are activated and the minimum and maximum threshold values that generate phosphene perception without causing retinal damage are determined for each electrode. Individualized stimulation programs are created and uploaded to the VPU. The glasses-mounted video microcamera is adjusted and positioned. Because the patient’s visual field is limited to 20° after implantation, they are taught how to recognize the position and shape of objects using head scanning movements;

- Rehabilitation process: One month after calibration, a long and difficult rehabilitation process using special methods under the supervision of specialists is required to teach artificial vision. This must be thoroughly explained to the patient and their family before implantation. At the end of the rehabilitation process, patients can distinguish the direction of movements; their orientation, mobility, and capacity for independent movement are improved; they can distinguish light and dark colors as shades of gray, see capital letters, and read short words; and their overall quality of life improves.^2,3,13^

## 4. Optimal candidates for Argus II retinal prosthesis

This epiretinal prosthesis is appropriate for patients with severe outer retinal degeneration but relatively spared inner retina. There must be a significant amount of viable RGCs in the inner retina to send electrical stimulation to the visual cortex and create the perception of phosphene.^14^ Therefore, this implantation is applicable in retinal degeneration patients with advanced RP, choroideremia, and advanced dry AMD. A preclinical study is also being conducted to test the feasibility and potential benefit of the system in GA.^15^

The patient’s and their family’s expectations from the implantation must be thoroughly understood and the potential benefits and limitations of the device must be accepted. Moreover, it is essential that the patient and their family be able and willing to follow through with the fitting and rehabilitation processes. Ophthalmologic examination findings in the patient must be within the appropriate range for implantation, which are:^13^

- The patient’s level of vision must be light perception, with positive camera flash test. If light perception is suspect, visual evoked response test must be normal,

- The patient should have the experience of seeing shapes prior to visual impairment,

- Because the implant currently in production is uniform with standard dimensions, in order for the array to be placed in the appropriate position on the macula, the patient must be at least 25 years of age and the anteroposterior length of the globe must be 20.5-26 mm.

- There must be no advanced strabismus or nystagmus that would disrupt the wireless communication between external coil and implanted coil.

- There must be no conjunctival and scleral disorders or macular staphyloma that would prevent the appropriate and secure implantation of system components.

- There must be no conditions that preclude the use of general anesthesia or the related medications.

## 5. Surgical Method

The eye is prepared as for pars plana vitrectomy with scleral buckling. After the silicone band including the receiver coil and electronics case is passed under the four rectus muscles, the ends of the band are connected with a Watzke sleeve at the superonasal quadrant. The electronics case is sutured to the superotemporal quadrant and the receiver coil is sutured to the inferotemporal quadrant under the lateral rectus according to the predetermined limbus distances. Following pars plana vitrectomy, total posterior hyaloid peeling and excision, and vitreous base removal, the electronic cable and epiretinal electrode array are inserted into the vitreous space through a 5.2-mm pars plana incision made about 3.5 mm from the limbus, and the array is positioned on the macula. After leak-proof suturing of the scleral incision, the array is placed on the macular region and is secured to the sclera using a tack that passes through a hole in the silicone matrix (Figures 3 and 4). At various stages of the surgery, impedance measurements are done using special computer systems in order to test whether any of the electronic components have been damaged. After the pars plana sclerotomies are closed, the electronics case and receiver coil are covered with pericardium, allograft sclera, or autologous aponeurosis to reduce conjunctival irritation and risk of erosion. Tenon’s capsule and the conjunctiva are sutured, and the procedure is concluded with intravitreal prophylactic antibiotic injection.^12,14,16,17^

## 6. Clinical Studies of Argus II

Phase 2 clinical studies on the feasibility and safety of this product were performed between 2007 and 2009. The poorer-seeing eyes of 30 patients aged 18-25 years who had previous history of useful form vision underwent implantation and the reliability of the implant was evaluated at 1 and 3 years.^14^ In 2014, analysis of 30 patients with a mean follow-up time of 6.2±0.9 years revealed that the implant had to be removed from 3 patients and was still functional in 24 of the remaining 27 patients.^2^

**Adverse effects, complications:** The Argus II epiretinal prosthesis system demonstrated an acceptable long-term safety profile and benefit 3 years after implantation. A total of 23 severe adverse effects occurred in 37% of the cases. Of these, 61% occurred within the first 6 months and 22% occurred more than 12 months after implantation. The commonest severe adverse effects were hypotonia, conjunctival erosion or dehiscence, and endophthalmia. All of these adverse effects could be treated with standard ophthalmic approaches. Using prophylactic intravitreal antibiotics at the end of the surgery and modifying the surgical technique and device designs significantly reduced the incidence of severe adverse effects.^13,14,18^

**Figure 5:**
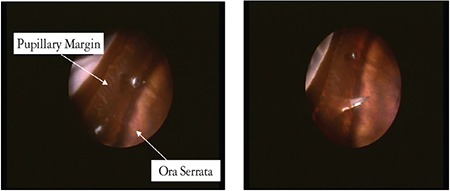
Endoscopic viewing of the retroiridal region during the implantation surgery to confirm the scleral incision site^19^

Anatomical variations in the ora serrata and pars plana are found in 47% of normal eyes. Therefore, severe complications such as choroidal/retinal detachment may occur during insertion of the array and electronic cable through the 5.2-mm scleral incision. These complications can be reduced with ophthalmic microendoscopic evaluation of the pars plana and ora serrata prior to scleral incision (Figure 5).^19^

**Visual function tests:** The level of vision produced with prostheses is not high enough for evaluation using standard visual function tests. Patients are objectively evaluated using specially designed computer-based low vision tests such as target localization (high-contrast square localization), direction of motion, grating visual acuity, letter recognition, orientation, and mobility tests. The performance rates detectable with these tests are generally higher when the Argus II epiretinal prosthesis system is in operation.^14^ Mean grating visual acuity test values at 1 and 3 years were 2.5 LogMAR and the best visual acuity was 1.8 LogMAR (20/1262 Snellen). Twenty-one patients performed the letter recognition test at a mean of 19.9 months after implantation. Letter groups organized according to certain properties were read correctly by 51.7-72.3% of the patients when the system was switched on and by 11.8-17.7% of the patients when the system was switched off. Six patients were able to read the smallest letter size of 0.9 cm from a distance of 30 cm.^2^ Orientation and mobility tests evaluate the patient’s performance in real-world conditions and includes indoor orientation tests, namely “door-finding” and “line-tracking”. With the system switched on, success rates in these tests were 54.2% and 67.9%, respectively, compared to 19% and 14.3% with the system switched off.^14^

**Figure 6:**
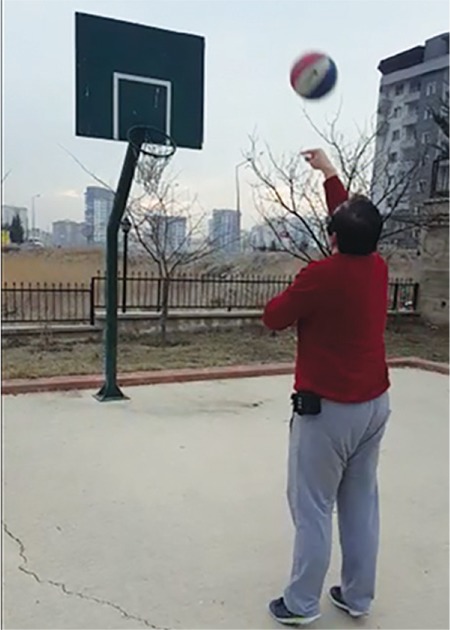
An end-stage retinitis pigmentosa patient able to perceive a basketball hoop 6 months after Argus II retinal prosthesis implantation; the black box at his waist is the Video Processing Unit (performed by surgeon E.Ö.)

**FLORA test (Functional Low-vision Observer-rated Assessment):** This test evaluates the effects of the Argus II retinal prosthesis in the patient’s everyday life. A visual rehabilitation specialist evaluated patients’ functional capabilities such as orientation, mobility, and social interaction during everyday life in the home environment at 1 month after implantation. The effect of the system on quality of life was rated as positive or moderately-positive in 80% of the patients at 1 year and 65.2% of the patients at 3 years (Figure 6).^2,3^

## 7. Problems with the Argus II System

No suitable and objective tests have been developed to determine the ideal candidate for implantation or to characterize the functional capacity of the residual retina. The effects of age, duration of low vision, and RP genotype on the outcomes of implantation are not fully known. However, the use of adaptive optics OCT in the near future should shed light on these problems.^2^

The long-term durability of the system’s electronic components and their long-term effects on the retina are also not fully known. As the microelectrode array and its connector cable remain in the intravitreal chemical environment for an extended period of time, there may be changes in their performance. In order to prevent damage to the degenerated retina during electrical stimulation, minimizing and appropriately dissipating the heat generated by the array is an important issue.

Patients must learn to use the phosphenes generated by electrical stimulation as visual information. The patient can localize objects within a 20° area using the camera mounted to the glasses. To do this, the patient must scan the environment with head movements instead of eye movements. This is a difficult process that must be learned.

**Figure 7:**
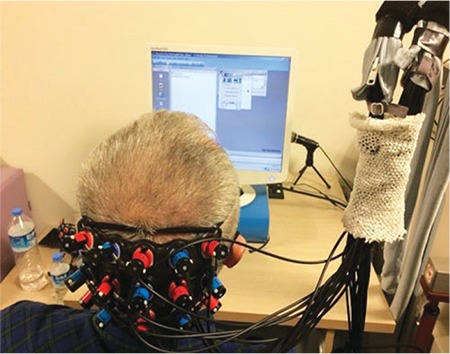
Functional near-infrared spectroscopy: a noninvasive optical method that provides information about cortical activity by measuring relative changes in oxy- and deoxyhemoglobin in the brain cortex upon stimulation of the visual cortex^20^

**Figure 8:**
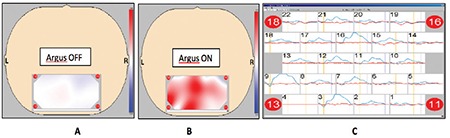
(A) There is ambiguous activity detected by functional near-infrared spectroscopy (fNIRS) in the occipital cortex when the Argus II system is nonoperational (Argus OFF). (B) Significant activity is detected by fNIRS in the occipital cortex when the Argus II system is operational (Argus ON). (C) Wave patterns recorded from the occipital cortex; blue wave: Argus ON, red wave: Argus OFF^20^

With functional MRI, it is possible to visualize the cortical areas that are activated and utilized during virtual vision tasks. However, the use of this method in patients with a metallic implant in their body involves various difficulties and disadvantages. These disadvantages can be eliminated by the development and use of functional near-infrared spectroscopy (fNIRS), which is easy to use, does not require a closed environment, and does not interact with metal implants. This would allow better postoperative functional assessment and more refined visual rehabilitation opportunities (Figures 7 and 8).^20,21^

## 8. Potential Developments Regarding the Argus-II

Considering the fact that there are millions of photoreceptors in a normal eye, the number of electrodes in the currently available arrays is very small. For this reason, patients do not yet have a general sense of their surroundings. Therefore, the current performance of the Argus II system must be increased.

- Developments in the glasses, VPU, battery, and digital camera design and the addition of eye-tracking, thermal perception, and depth information would improve the current system.

- Advances in the software and the image and signal processing algorithms can further enhance visual perception, orientation, and mobility without changing the existing hardware.^22^ It is also possible to improve visual perception and widen the visual field through advances in the hardware.^23^

- Magnification and minimization of the acquired image may further enhance visual acuity. The image can be adjusted between 0.4x and 16x with a hand-held controller. A vision level equivalent to 20/200 was obtained in grating visual acuity test at 16x magnification and letters 2.3 cm in size were read from 30 cm distance with 4x magnification.^24^

- The addition of a face recognition algorithm to the system may enable face localization from a distance of 2-3 m.^25^

- With the introduction of three-dimensional needle electrodes, it will be possible to stimulate the retinal neural network while also evaluating the electrochemical events occurring within the retina in real time.

## Conclusion

Studies on the Argus II epiretinal prosthesis performed to date have demonstrated the long-term safety and potential benefits of controlled chronic electrical stimulation in patients with advanced visual impairment due to outer retinal degeneration associated with conditions such as RP, choroideremia, and GA. Some of the missing pieces in information obtained through artificial vision are filled in by the brain based on previous experiences. The limited number of clinical studies performed with various other retinal prostheses other than the Argus II system have also yielded promising results. However, each type of prosthesis has its own advantages and disadvantages. Cortical implants, which are currently in the preclinical study phase, may provide artificial vision to patients with complete retina and optic nerve destruction.
